# Determination of Biochemical and Metabolomic Characteristics of Sheep Blood Serum and Their Application in Clinical Practice

**DOI:** 10.3390/life15071141

**Published:** 2025-07-20

**Authors:** Peter Očenáš, Matej Baloga, Marcela Valko-Rokytovská, Sonja Ivašková

**Affiliations:** 1 Department of Chemistry, Biochemistry and Biophysics, University of Veterinary Medicine and Pharmacy in Košice, Komenského 73, 041 81 Košice, Slovakia; matej.baloga@gmail.com (M.B.); marcela.valko-rokytovska@uvlf.sk (M.V.-R.); 2 Department of Morphological Disciplines, University of Veterinary Medicine and Pharmacy in Košice, Komenského 73, 041 81 Košice, Slovakia; sonja.ivaskova@student.uvlf.sk

**Keywords:** blood serum, biomarkers, biochemical analysis, HPLC, metabolomics

## Abstract

Due to advances in molecular technologies and the expanding knowledge of biomarkers, their use in patient screening, diagnosis, prognosis, and targeted therapy is continuously increasing. Biomarker characteristics play a crucial role across all areas of medical research/practice. Biomarkers often reflect changes in the biochemical composition of biofluids, which can be qualitatively and quantitatively analyzed using methods such as high-performance liquid chromatography (HPLC) at various stages of clinical intervention. This study focuses on establishing physiological reference ranges for selected biochemical and metabolomic indicators by analyzing blood serum samples from domestic sheep. A total of sixty samples are examined using standard biochemical assays and HPLC, resulting in the determination of experimental reference values for twenty-one biochemical and eight metabolomic parameters. Reliable and reproducible preclinical testing is essential before any diagnostic method can be introduced into clinical use. A thorough understanding of the safety and efficacy of such methods in animal models is a prerequisite for initiating human trials. Species selection and the definition of physiological biomarker ranges are therefore critical components in the development of effective preclinical protocols. This work contributes to the foundation needed for further clinical testing by establishing reference values for relevant biomarkers in a commonly used animal model.

## 1. Introduction

In the long history of medical research, animals have always been important investigation tools. Determination of the biochemical, mineral, and enzymatic profile of the blood serum in an animal model is a key step in monitoring the physiological and pathological processes. Biochemical data obtained from clinical cases need to be evaluated and compared with the available reference intervals of individual blood parameters. Determining reference intervals is acknowledged to be difficult, time-consuming, and costly. The value of any research project is fundamentally dependent on the validity and reproducibility of the data [1].

Biochemical analysis and metabolomics are processes that are closely related, complementing each other, yet differing in their approach. Biochemical analysis examines specific metabolites (e.g., glucose, urea, aspartate aminotransferase, calcium) in biological fluids using standard clinical tests (e.g., spectrophotometry, enzymatic methods) with the aim of determining the physiological or pathological state of the organism. It is a targeted analysis that monitors only a few selected parameters. Metabolomics, on the other hand, is a much broader and more modern approach. It analyzes hundreds to thousands of metabolites simultaneously using more advanced techniques such as mass spectrometry (MS), nuclear magnetic resonance spectroscopy (NMR), and liquid/gas chromatography (LC-MS, GC-MS). Its goal is to create a comprehensive picture of the organism’s metabolic profile (the so-called metabolome). It is used for biomarker discovery, the study of diseases, nutrition, stress, or treatment effects. Metabolomics is a non-targeted or semi-targeted analysis aimed at gaining a global understanding of metabolic processes [2].

Despite ongoing debates regarding the use of animal models in research, their importance in biomedical science and regenerative medicine is undeniable. In vivo studies serve as a critical bridge between in vitro research and clinical applications. Due to their anatomical and physiological similarities to humans, animal models such as mice, rats, rabbits, pigs, goats, dogs, and sheep are frequently used in preclinical testing. To evaluate pathological processes and monitor transplant outcomes effectively, it is essential to establish baseline physiological values of key biochemical and metabolomic markers.

**Figure 1:**
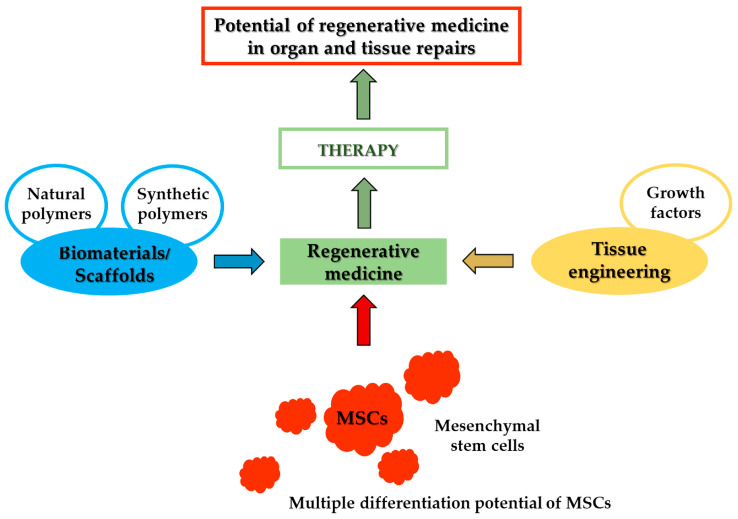
General procedures in the development of regenerative medicine.

The rapid advancement of modern diagnostic methods in medicine significantly contributes to research aimed at understanding the pathological processes of various diseases. One of the most dynamically developing fields is regenerative medicine, which seeks to replace or regenerate human cells, tissues, and organs in order to restore or establish normal function (Figure 1). The period surrounding transplantation is particularly demanding for patients—not only physically, but also psychologically and socially. Regenerative procedures involve the introduction of foreign materials or tissues into the body, which must be accepted and integrated by the host organism. Although modern transplant techniques utilize biocompatible materials, thorough post-transplant monitoring remains essential [3,4,5,6].

**Figure 2:**
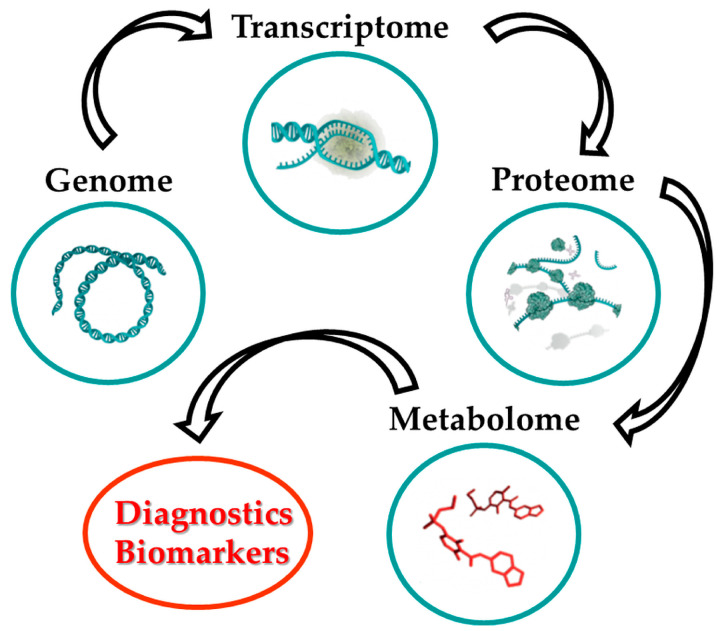
Metabolomics as a diagnostic indicator.

The healing process after transplantation is long and requires careful monitoring. In this context, metabolomic approaches are increasingly used to assess physiological changes. Metabolomics is a multi-analytical method focused on identifying and quantifying low molecular weight metabolites present in biological samples under defined conditions [7,8]. These metabolites form the so-called metabolome, which reflects both the genetic makeup and the dynamic physiological state of the organism [9]. Comprehensive metabolomic analysis often requires a combination of different analytical techniques due to the chemical diversity of metabolites (Figure 2).

However, data on biochemical and metabolomic reference values in Slovak domestic sheep (*Ovis aries*) are limited. Although some studies provide selected biochemical parameters in sheep serum [10,11,12], only a few report concentrations of free amino acids [13,14,15]. Changes in serum proteins can indicate disease progression or early post-operative complications, such as infections or inflammation [16]. Tóthová et al. examined acute phase proteins in sheep after experimental cartilage reconstruction [5]. However, our study expands upon previous work by analyzing a broader panel of parameters.

In this study, we established physiological reference ranges for selected biochemical markers (e.g., albumin, alkaline phosphatase, alanine aminotransferase, aspartate aminotransferase, blood urea nitrogen, creatinine, γ-glutamyltransferase, glucose, total bilirubin, total cholesterol, total protein, uric acid, globulin, urea, albumin/globulin ratio, calcium, chloride, potassium, sodium, phosphorus), as well as for C-reactive protein and selected metabolites (creatinine, tyrosine, tryptophan, methionine, hydroxyproline, adenosine, hypoxanthine, neopterin). These values were obtained from serum samples of Slovak domestic sheep using biochemical and ultra-high performance liquid chromatography (UHPLC) analysis. The findings aim to support the interpretation of preclinical results and improve our understanding of biochemical changes during the pre- and post-transplantation phases, particularly in cartilage and bone tissue engineering.

## 2. Materials and Methods

### 2.1. Sample Collection

All animal procedures were approved by the State Veterinary and Food Administration of the Slovak Republic No. 2220/17-221. Blood samples were collected on October 2023 from 60 healthy sheep (all females), improved Wallachian breed, aged from 2 to 5 years. The sheep in extensive farming system on green pastures throughout the whole year were raised, except in winter when they were kept in a barn. They also had constant access to fresh water and hay. All sheep in good health, regularly vaccinated and dewormed were kept. Each sheep breeding facility holds a “Health Status” certificate issued by the respective Regional Veterinary Authority. These facilities are regularly inspected once a month by an official veterinarian, who checks both the animals’ health condition and their welfare. In the event of a disease outbreak, inspections are carried out more frequently, depending on the specific disease. Blood samples were taken by a veterinarian, who thoroughly examined the health condition of each animal prior to sampling. Pregnancy was not confirmed.

### 2.2. Blood Sampling and Serum Preparation

The blood was aseptically collected via jugular venepuncture (*v. jugularis externa*). The jugular groove was disinfected using either iodopovidone (Betadine 100 mg × mL^−1^, Egis Pharmaceuticals PLC, Budapest, HU) or chlorhexidine (SkinMed spray, Cymedica SK, spol. s.r.o., Zvolen, SK). Whole blood (4.4 mL) was collected into serum activator tubes (Serum Gel Z/4.4 mL, SARSTEDT spol. s.r.o., Bratislava, SK) and left to clot for 30 min at room temperature in an upright position. Subsequently, the samples were centrifuged using a Benchmark LC-8 centrifuge (Benchmark Scientific, Inc., Sayreville, NJ, USA) at 5000 rpm for 5 min to separate the serum. The sera were then transferred into Eppendorf tubes and stored at −42 °C until further analysis.

### 2.3. Preparation of Mobile Phase for HPLC Analysis

The target HPLC grade analytes (Sigma-Aldrich, Bratislava, Slovakia) creatinine, tyrosine, tryptophan, methionine, hydroxyproline, adenosine, hypoxanthine, and neopterin were determined in blood samples using UHPLC analysis under gradient conditions with a two-component mobile phase: (A) methanol and (B) water with 0.1% formic acid, mixed in a predetermined ratio. Component A was a commercially available methanol (HPLC grade, Fisher Chemical, Waltham, MA, USA) and Component B was prepared by mixing 250 mL of deionized water (HPLC grade, Fisher Chemical) with 250 µL of formic acid (HPLC grade, Sigma-Aldrich) in a volumetric flask. Prior to analysis, the freshly prepared mobile phase was placed in an ultrasonic bath (30 min, Bandelin Sonorex, Berlin, Germany) to ensure complete degassing.

### 2.4. Preparation of Stock Standard Solutions

Fresh stock solutions with a concentration of 1000 µg × mL^−1^ were prepared by dissolving 1 mg of each selected analyte in 1 mL of deionized water and vortexing for 1 min. The required calibration concentrations were obtained by serial dilution of these stock solutions. Eight calibration curves were constructed from the prepared standard solutions. The calibration curve (Lin, AddZero) for creatinine, tyrosine, tryptophan, methionine, hydroxyproline, adenosine, hypoxanthine, and neopterin consisted of seven concentration points: 50; 25; 10; 5; 1; 0.1; 0.01 µg × mL^−1^.

### 2.5. Preparation of Samples for Biochemical and UHPLC Analysis

Blood serum samples from domestic sheep were stored in a freezer at a constant temperature of −42 °C until analysis. Prior to processing, the samples were thawed and vortexed for 1 min. The serum was used for biochemical analysis without any further modification. For UHPLC determination, the methanol was added to the serum sample in a 1:3 ratio (sample/methanol) to precipitate proteins. The entire volume of the precipitated sample was then centrifuged for 20 min at 5000 rpm using an Eppendorf Centrifuge 5430. The resulting supernatant was carefully collected without disturbing the pellet and filtered through PVDF Frisenette Q-max RR Syringe filters (25 mm; 0.22 µm). Finally, the processed blood serum sample was injected into the UHPLC system for analysis.

### 2.6. Biochemical Analysis of Blood Serum

Biochemical analysis of domestic sheep blood serum samples was carried out using the Exdia TRF plus and Skyla HB1 biochemical analyzers (Axon Lab spol. s r.o., Prague, Czech Republic). The Exdia TRF plus biochemical analyzer was used to determine C-reactive protein (CRP). For broader biochemical analysis, the Skyla HB1 analyzer was employed with two diagnostic panels. The general biochemistry panel included fifteen clinical indicators: ALB (albumin), ALP (alkaline phosphatase), ALT (alanine aminotransferase), AST (aspartate aminotransferase), BUN (blood urea nitrogen), CREA (serum creatinine), GGT (γ-glutamyltransferase), GLU (glucose), TBIL (total bilirubin), TC (total cholesterol), TP (total protein), UA (uric acid), GLOB (globulin), UREA (urea), A/G ratio (albumin/globulin ratio). The metabolic panel included sixteen clinical indicators: ALB, ALT, AST, BUN, Ca (calcium), Cl (chloride), CREA, GLU, K (potassium), Na (sodium), PHOS (phosphorus), TP, UA, A/G ratio, GLOB and UREA. Each analysis was performed by applying 200 µL of a blood serum to the appropriate test disc. Prior to analysis, the discs were stored in a refrigerator at a temperature of 2–8 °C. The analysis of each sample took approximately 15 min after placing the disc into the analyzer.

### 2.7. Qualitative and Quantitative UHPLC Analysis of Blood Serum

Potential markers (creatinine, tyrosine, tryptophan, methionine, hydroxyproline, adenosine, hypoxanthine, and neopterin) were analyzed using a Dionex UltiMate 3000 RS UHPLC system (Thermo Fisher Scientific, Massachusetts, USA). Separation of the selected analytes was performed on a YMC-Triart PFP chromatography column (150 × 3.0 mm I.D., S-1.9 µm, 12 nm, YMC Europe GmbH, Dinslaken, Germany). The column temperature was maintained at 30 °C with an accuracy of ±0.5 °C throughout the 20 min analysis. The injection volume for standards and samples was set to 10 μL. The mobile phase consisted of two components: methanol (A) and deionized water with 0.1% formic acid (B). Separation was carried out under gradient conditions, with the proportion of Component B changing over time as follows: 100–70% B (0–15 min), 70–100% B (15–15.1 min), 100% B (15.1–20 min). The flow rate was set to 0.5 mL/min. Analytes were detected using serially connected detectors diode array detector (DAD, λ = 200/220 nm) and fluorescence detector (FLD, λ_ex_ = 280 nm and λ_em_ = 350 nm). Chromatographic data were processed and evaluated using Chromeleon 7.2 Chromatography Data System software.

## 3. Results

In the presented study, an analysis of biological material (blood serum) was performed. This research work focuses on the creation of physiological ranges of selected parameters using the described biochemical and UHPLC analysis for the upcoming evaluation of the biochemical-metabolomics profile of a set of blood serum samples in the pre- and post-transplantation periods. The use of biofluids as a medium for the study of targeted metabolomics is considered the less/more invasive, since the provision of samples represents the intervention in the patient’s organism. More invasive sampling methods than urine samples are used to collect blood and synovial fluid samples. The diversity of analyzed biomaterials increases the diagnostic value of monitored diseases. Newer studies deal with the progress and severity of the given diseases while also investigating the bioactive factors in connection with bone/cartilage degradation [17].

The chosen animal model, the domestic sheep, is suitable for studying the biochemical–metabolomic profile of biological fluids, as physiological processes take place similarly to humans, and therefore we can consider it an adequate substitute for preclinical studies. Sheep models possess body weights similar to human adults, akin turnover and remodeling of organism, and possess longer/larger tissues with comparable microstructure, despite several reported histological differences.

A set of 60 control blood serum samples of a Slovak domestic sheep as a research tool was used. Clinical biochemical analysis and subsequent determination of concentrations of blood parameters and enzyme activities is currently an essential diagnostic aid. The specific activity of enzymes and their presence in different tissues points to their use as a diagnostic marker.

First, biochemical analysis of healthy control blood serum samples was performed. Fifteen and sixteen clinical-biochemical in vitro diagnostic indicators by biochemical analysis of a set of 60 blood serum samples using the Skyla HB1 analyzer with general biochemistry panel (ALB, ALP, ALT, AST, BUN, CREA, GGT, GLU, TBIL, TC, TP, UA, GLOB UREA, A/G ratio) and metabolic panel (ALB, ALT, AST, BUN, Ca, Cl, CREA, GLU, K, Na, PHOS, TP, UA, A/G ratio, GLOB) were determined. An Exdia TRF plus biochemical analyzer to determine C-reactive protein was used. Two different panels were used in the analysis to expand the biochemical profile of sheep blood serum. Their combination was used to define the comprehensive biochemical, mineral, and enzymatic status of the sheep. All blood serum control samples in duplicate to allow accurate interpretation of the resulting concentrations were analyzed. Clinical parameters were evaluated in multiples of the unit g × L^−1^, mmol × L^−1^ or, in the case of enzymes (ALP, ALT, AST and GGT), their activity was evaluated, which was reported in units of U × L^−1^, where U represents the conversion of 1 µmol of substrate in 1 min. The concentration values UA are listed in Table 1 with the appropriate mathematical sign (<), as they were lower than the lowest measurable concentration declared by the analyzer. After the analysis of all blood serum control samples, experimental average values with standard deviations (±SDs) and physiological ranges of individual biochemical parameters by mathematical calculation were determined. The experimentally determined concentration values are listed in Table 1. The normality of distribution for the measured biochemical parameters was assessed using the D’Agostino and Pearson omnibus test (α = 0.05). Seven parameters (TP, GLU, TC, CREA, PHOS, K, and Cl) did not significantly deviate from a normal distribution (*p >* 0.05), whereas the remaining parameters (ALB, ALP, ALT, AST, GGT, BUN, Ca, Na, GLOB, A:G, UREA and CRP) showed significant deviation from normality (*p <* 0.05). Data for uric acid (UA) could not be evaluated due to invalid input. The obtained results were used to compare control samples with pathological ones and to monitor possible fluctuations in biochemical processes in the organism.

**Figure 3:**
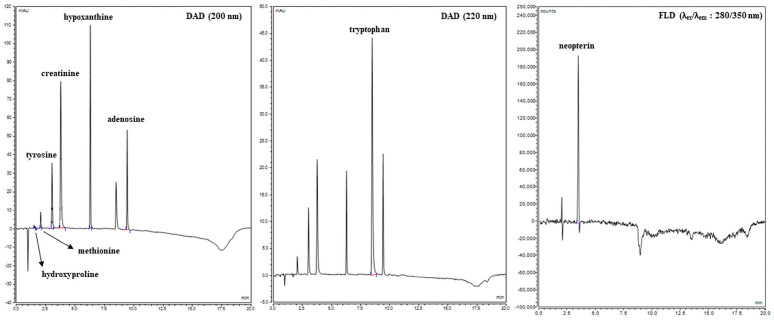
Chromatogram of selected metabolites.

UHPLC analysis of eight targeted metabolites (creatinine, tyrosine, tryptophan, methionine, hydroxyproline, neopterin, adenosine, and hypoxanthin) in blood serum using serially connected DAD and FLD detectors was performed. The wavelengths for DAD (λ = 200/220 nm) and FLD (λ_ex_ = 280 nm and λ_em_ = 350 nm) detection were chosen according to the original study [18]. Retention times were assigned to individual separated analytes after optimization of the UHPLC method, which were on average creatinine (3.537 min), tyrosine (3.027 min), tryptophan (8.493 min), methionine (2.070 min), hydroxyproline (1.527 min), neopterin (3.390 min), adenosine (9.423 min), and hypoxanthin (6.283 min, Table 2). Average values of retention times were obtained by analyzing standards with a concentration of 10 µg × mL^−1^ (Figure 3). Calibration curves of the selected metabolites were constructed based on the relationship between the generated peak area values and the appropriately chosen concentrations. The calibration curves for quantification of the selected analytes included seven concentration points (50; 25; 10; 5; 1; 0.1; 0.01 µg × mL^−1^). All calibration solutions were analyzed in duplicate to ensure maximum accuracy, and the corresponding linear equations and correlation coefficients were calculated. Qualitative identification of the selected metabolites in the samples was performed by assigning retention times to the respective signals based on characterized standards. Quantitative analysis of the selected metabolites in the samples was subsequently carried out by integrating the identified signals and applying the results to the established calibration curves. The precision of the method was evaluated by intra- and interday tests. Precision and recovery were determined by duplicate analysis of the blood samples in which the values of examined metabolites were calculated on three consecutive days (Table 2).

Since targeted monitoring of metabolites in the blood serum of domestic sheep and physiological values of selected metabolites is low, as described in scientific works, it was necessary to analyze and evaluate all control samples. Subsequently, after obtaining the quantitative data, the experimental average values with the standard deviations (±SDs) and the physiological ranges of the individual metabolites by mathematical calculation were determined. The experimentally determined concentration values are shown in Table 3. The obtained results are necessary for comparing control samples with pathological ones and for later monitoring of possible fluctuations during the transplantation process.

## 4. Discussion

To date, only a limited number of studies have explored the use of biomarkers as diagnostic indicators in the context of bone and joint biopolymer substitutes. Existing research has predominantly focused on inflammation-related biomolecules, bone degradation markers, and immune response factors, primarily within animal models. Although several molecules have shown diagnostic potential, opportunities remain for the discovery of novel, more specific biomarkers.

The present study analyzed blood serum samples from 60 healthy Slovak sheep to determine experimental reference values for 21 biochemical parameters. These baseline values are intended to support future studies that will monitor changes in biomarker concentrations at various stages of surgical transplantation—preoperative, intraoperative, and postoperative. This study provides a biochemical reference profile in healthy sheep, laying the groundwork for future research into biomarker-based monitoring in orthopedic transplantation models.

### 4.1. Biochemical Parameters and Enzyme Activities in Healthy Sheep

Biochemical analysis of body fluids is a primary method in the study of physiological and pathological conditions. Several biochemical parameters are used as key, specific indicators in evaluating the course or treatment of a disease. In the context of our scientific research, ALP (mineralization of bone tissue), Ca, and PHOS (bone biosynthesis and degradation) are considered potential markers. Of course, the other selected parameters also significantly reflect the overall course of physiological/pathological/metabolic processes (inflammatory response and immune system activation-CRP, stress reactions, glucose, fat and lipid metabolism, kidney and liver dysfunction-CREAT, ALT, AST, TBIL, etc.).

Biochemical parameters, including enzyme activities, were measured using the Skyla HB1 analyzer. Each sample underwent duplicate analysis to ensure accuracy. Experimentally determined physiological values of biochemical parameters in blood serum together with standard deviations (SDs) are presented in Table 1. The physiological ranges for ALP (124.0–466.0 U × L^−1^), ALT (17.0–30.0 U × L^−1^), AST (104.0–156.0 U × L^−1^), and GGT (45.0–75.0 U × L^−1^) were established. Comparing our results with the available studies [10,19,20], we can evaluate that the biochemical and mineral profile of the blood serum of our domestic sheep determined by us was slightly different. Sarmin et al. present in their study [10] the biochemical, mineral, and enzymatic profile of sheep blood serum in different physiological states (ram, pregnant, suckling lamb, etc.). When comparing the results of the analyzed parameters, we found that our values are comparable for the parameters ALP (274.97–581.94 U × L^−1^), CREA (0.76–1.45 mg × dL^−1^), TBIL (0.40–0.48 mg × dL^−1^), UREA (26.60–68.80 mg × dL^−1^), Ca (9.48–10.96 mmol × L^−1^), and Cl (101.33–110.83 mmol × L^−1^). Slightly lower concentration values compared to ours were observed for TP (6.73–7.26 g × dL^−1^), GLOB (2.53–4.01 g × dL^−1^), and K (3.13–4.09 mmol × L^−1^), whereas slightly higher values were noted for ALB (3.66 g × dL^−1^), A/G (0.62–1.20), and Na (142.33–153.83 mmol × L^−1^). In the case of the study by Eschbach et al. [19], we observed slight deviations in serum concentrations for ALB (2.2 g × dL^−1^), K (4.6 mmol × L^−1^), Ca (9.3 mmol × L^−1^), and Na (146 mmol × L^−1^), and more significant deviations for ALP (57.0 U × L^−1^). In another study [20], differences were observed in the form of decreased serum levels of CREAT (47.20–66.10 μmol × L^−1^), Ca (1.97–2.51 mmol × L^−1^), and PHOS (1.23–2.02 mmol × L^−1^), and increased levels of GLOB (32.90–47.10) and GGT (19.80–64.50 U × L^−1^). Species and geographical variations (Indonesia/USA/Croatia vs. Slovakia) are among the primary factors that may have contributed to the observed differences in serum concentrations. Serum levels are also significantly influenced by factors such as age, sex, reproductive status, diet/nutrition, stress, seasonality, and climatic conditions.

### 4.2. Inflammatory Markers

Reference CRP levels were established in healthy sheep (0.262 ± 0.058 mg × L^−1^), offering a foundation for detecting deviations associated with pathological or post-surgical conditions. A 2019 study monitored total serum proteins (TSPs) and acute-phase proteins—serum amyloid A (SAA), haptoglobin (Hp), and CRP—in sheep undergoing experimental cartilage transplantation. Slight fluctuations in TSP and transient increases in α-globulin fractions were observed around day 14 post operation, with a return to baseline by day 30. Elevated CRP levels prior to surgery further increased postoperatively but normalized by the second week. The inflammatory response following biopolymer implantation in sheep was moderate and self-resolving, demonstrating the potential for specific markers to monitor postoperative recovery [5]. Another study assessed inflammation in pigs following cartilage defect repair using different formulations of tetracalcium phosphate cement (C-cement). All experimental groups showed elevated inflammatory markers at day 7, followed by a gradual decline to preoperative levels by day 30, indicating a normal healing response. Notably, C-cement without amino acid enrichment elicited a milder inflammatory reaction [21]. A separate study with amino acid-enriched C-cement (CAK) demonstrated similar trends in pigs: increased levels of SAA, Hp, CRP, and pig-MAP peaking at day 7 and declining thereafter. However, α1-acid glycoprotein (AGP) levels remained largely unchanged, suggesting limited diagnostic value. Enzymatic markers such as creatine kinase (CK) and lactate dehydrogenase (LD) reflected muscle and tissue injury, while alkaline phosphatase (AP) activity initially decreased and later rebounded [22]. Inflammation-related biomarkers proved effective in tracking postoperative tissue responses in porcine models, with material composition influencing the immune reaction.

### 4.3. Metabolite Profiling Using UHPLC

In the second phase of the study, key metabolites were analyzed using an UHPLC-DAD-FLD system. Targeted molecules included creatinine, selected amino acids (tyrosine, tryptophan, methionine, hydroxyproline), and purine-related compounds (adenosine, hypoxanthine, neopterin). These compounds are closely linked to bone metabolism, oxidative stress, and immune responses. Advanced chromatographic techniques enabled precise quantification of biologically relevant metabolites, further supporting their use as diagnostic tools in orthopedic research.

### 4.4. Creatinine as a Marker of Renal and Bone Function

Creatinine concentrations in sheep ranged from 0.070 to 0.100 mmol × L^−1^, aligning with physiological values reported in other species [11,23,24,25] and comparable to human reference ranges [26]. Previous studies have confirmed that creatinine is not only a reliable indicator of renal function [27] but may also serve in bone-related diagnostics through its ratio with albumin (albumin/creatinine ratio-ACR) or hydroxyproline [28,29]. Creatinine remains a robust biomarker of systemic function, with potential applications in evaluating bone turnover and postoperative metabolic shifts.

### 4.5. Amino Acids and Bone Metabolism

**Figure 4:**
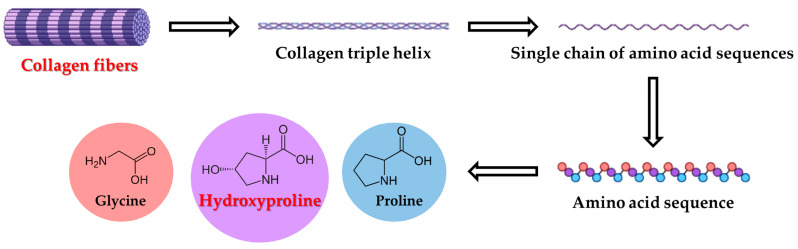
Degradation of collagen fibers.

Tyrosine and tryptophan, due to their susceptibility to oxidation, are implicated in oxidative stress-related conditions [14,30,31,32,33,34,35,36,37,38,39]. Oxidized forms of these amino acids may interfere with bone cell proliferation and differentiation [17,40,41,42]. Methionine, an essential amino acid, contributes to osteoblast function and epigenetic regulation [43,44,45,46]. Hydroxyproline, a collagen-specific amino acid (Figure 4), is a well-established marker of bone resorption and extracellular matrix turnover [47,48,49,50]. The physiological levels of selected amino acids are shown in Table 3. The mean values of tyrosine (92 ± 27 µmol × L^−1^), tryptophan (57 ± 26 µmol × L^−1^), methionine (41 ± 12 µmol × L^−1^), and hydroxyproline (34 ± 10 µmol × L^−1^) were calculated. Compared to previous studies [14,30], where amino acids reached concentrations 88.7 µmol × L^−1^ for tyrosine, 65.6 µmol × L^−1^ for tryptophan, 34.5 µmol × L^−1^ for methionine, and 13.2 µmol × L^−1^ for hydroxyproline, our results represent slightly increased concentration values except the value for tryptophan. Amino acid profiles, particularly of tyrosine, tryptophan, methionine, and hydroxyproline, provide valuable insight into bone metabolism and oxidative damage, with implications for orthopedic monitoring.

### 4.6. Purine Metabolites and Immune System Activation

Adenosine levels (1.2–2.4 µmol × L^−1^) increase in response to hypoxic or inflammatory stimuli [51,52,53]. Hypoxanthine (2.2–4.1 µmol × L^−1^), a downstream product of purine metabolism, is linked to oxidative stress and ischemia-reperfusion injury [54]. These metabolites offer dynamic biomarkers of cellular stress and energy imbalance. Adenosine and hypoxanthine represent sensitive markers of hypoxia and metabolic stress relevant to tissue injury and healing.

Neopterin levels (2.3–3.7 µmol × L^−1^) were measured as indicators of immune system activation. Although elevated in several inflammatory conditions, neopterin correlation with disease activity remains inconsistent across studies [55,56]. Neopterin may serve as a general immune marker, though its diagnostic precision in orthopedic applications requires further validation.

The management of musculoskeletal disorders frequently involves surgical intervention and transplantation, processes that trigger a cascade of biochemical and metabolic responses. This study presents experimental reference values for key biochemical parameters and metabolites in sheep serum, laying the foundation for future translational research. Identifying reliable biomarkers for bone and joint regeneration remains a crucial goal for improving diagnostic accuracy and therapeutic outcomes. This work contributes essential baseline data for biomarker development, offering new avenues for monitoring tissue healing and evaluating biopolymer transplant success.

## 5. Conclusions

Currently, in human medicine, we encounter a growing number of diseases of the musculoskeletal system of various etiologies. Many of them often require surgery or transplantation, and it is during this process that a number of metabolic changes take place in the body, manifested by deviations of selected biomarker levels. In the article, sixty sheep blood serum samples (healthy controls) as animal model were analyzed and experimental physiological values of biochemical parameters and selected biomarker for next clinical research were created. Biomarkers with a higher prognostic potential are being constantly sought in an effort to improve the pharmacological and surgical therapy of patients. A large number of them, pointing to metabolic changes of the connective tissue, have been already identified, but it is still necessary to study and characterize specific diagnostically significant biomolecules.

## Figures and Tables

**Table 1:** Physiological blood serum biochemistry values of domestic sheep.

**Clinical-chemical in vitro diagnostic parameters**	**Experimental Physiological Values of Selected Biochemical Parameters** **(Samples, *n* = 60)**						
			**Mean ± SD**	**Median; IQR**	**Minimum**	**Maximum**	**Physiological**
	**SKYLA**	**TP** (g × dL^−1^)	8.26 ± 0.630	8.3; 0.7	6.7	10.0	7.6–8.9
		**ALB** (g × dL^−1^)	3.13 ± 0.343	3.1; 0.2	2.1	4.9	2.8–3.5
		**GLOB** (g × dL^−1^)	5.13 ± 0.393	5.2; 0.675	3.5	7.2	4.7–5.6
		**A/G**	0.62 ± 0.099	0.6; 0.2	0.3	1.0	0.5–0.8
		**ALP** (U × L^−1^)	294.9 ± 170.418	282.0; 301.0	76.0	605.0	124.0–466.0
		**ALT** (U × L^−1^)	23.3 ± 6.283	20.0; 5.0	20.0	48.0	17.0–30.0
		**AST** (U × L^−1^)	130.2 ± 25.718	134.0; 20.5	84.0	209.0	104.0–156.0
		**GGT** (U × L^−1^)	60.5 ± 14.827	59.0; 12.0	38.0	117.0	45.0–75.0
		**CREA** (mg × dL^−1^)	1.1 ± 0.129	1.055; 0.19	0.8	1.4	0.9–1.3
		**TBIL** (mg × dL^−1^)	0.4 ± 0.057	0.4; 0.0	0.32	0.48	0.35–0.45
		**TC** (g × dL^−1^)	83.8 ± 18.432	79.5; 17.0	55.0	115.0	65.0–103.0
		**GLU** (mg × dL^−1^)	44.8 ± 8.400	45.0; 11.0	30.0	68.0	36.0–53.5
		**UA** (mg × dL^−1^)	<1.0	<1.0	<1.0	<1.0	<1.0
		**UREA** (mg × dL^−1^)	49.6 ± 8.087	47.3; 9.6	33.6	68.1	41.5–58.0
		**BUN** (mg × dL^−1^)	23.2 ± 3.778	22.1; 4.5	15.7	31.8	19.0–27.0
		**Na** (mmol × L^−1^)	140.6 ± 5.875	141.5; 6.0	120.0	153.0	134.5–146.5
		**K** (mmol × L^−1^)	5.2 ± 0.455	5.15; 0.7	4.2	5.9	4.7–5.7
		**Ca** (mmol × L^−1^)	10.4 ± 0.626	10.4; 0.6	8.6	11.3	9.7–11.3
		**Cl** (mmol × L^−1^)	106.9 ± 3.673	104.5; 10.0	89.0	112.0	103.2–110.6
		**PHOS** (mg × dL^−1^)	5.8 ± 0.953	5.6; 1.5	3.9	7.6	4.8–6.8
	**EXDIA**	**CRP** (mg × L^−1^)	0.262 ± 0.058	0.25; 0.04	0.18	<0.38	0.20–0.32

TP (total protein), ALB (albumin), GLOB (globulin), A/G ratio (albumin/globulin ratio), ALP (alkaline phosphatase), ALT (alanine aminotransferase), AST (aspartate aminotransferase), GGT (γ-glutamyltransferase), CREA (serum creatinine), TBIL (total bilirubin), TC (total cholesterol), GLU (glucose), UA (uric acid), UREA (urea), BUN (blood urea nitrogen), Na (sodium), K (potassium), Ca (calcium), Cl (chloride), PHOS (phosphorus), CRP (C-reactive protein), SD (standard deviation), IQR (interquartile range).

**Table 2:** Overview of retention times and calibration parameters of selected analytes.

**Analytes**	**Detection**	**Retention** **Times** **(t_R_, min)**	**Calibration Line** **Equation**	**Correlation** **Coefficient** **(R^2^)**	**RSD** **(%)**	**Recovery** **(Intra/Inter, %)**	**LOD** **(ng × mL^−1^)**	**LOQ** **(ng × mL^−1^)**
**creatinine**	DAD	3.537	x = 0.7067y + 0.0714	0.9998	2.516	103.1/107.2	7	22
**tyrosine**	DAD	3.027	x = 0.2456y + 0.0082	0.9998	5.459	102.3/104.2	8	25
**tryptophan**	DAD	8.493	x = 0.4355y − 0.0191	0.9998	4.980	99.0/101.0	5	15
**methionine**	DAD	2.070	x = 0.0501y − 0.0022	0.9978	3.152	101.0/105.0	9	28
**hydroxyproline**	DAD	1.527	x = 0.0714y	0.9966	6.728	98.0/102.0	10	31
**adenosine**	DAD	9.423	x = 0.3078y − 0.0085	0.9993	4.239	101.0/103.0	7	23
**hypoxanthin**	DAD	6.283	x = 0.5911y + 0.0839	0.9991	3.855	99.0/102.0	6	20
**neopterin**	FLD	3.390	x = 1933.3816y + 353.221	0.9995	2.791	101.0/103.0	5	17

RSD (relative standard deviation), LOD (limit of detection), LOQ (limit of quantification), DAD (diode array detector), FLD (fluorescence detector).

**Table 3:** Physiological blood serum metabolomics values of domestic sheep.

Experimental Blood Serum Values of Selected Metabolomics Parameters [Samples (*n* = 60); µmol × L^−1^]					
Metabolites		Mean ± SD	Minimum	Maximum	Physiological
**UHPLC**	**creatinine**	88 ± 17	54	117	70–100
	**tyrosine**	92 ± 27	11	143	65–120
	**tryptophan**	57 ± 26	20	102	30–85
	**methionine**	41 ± 12	19	74	29–55
	**hydroxyproline**	34 ± 10	16	59	24–45
	**adenosine**	1.795 ± 0.560	1.022	3.182	1.2–2.4
	**hypoxanthin**	3.150 ± 0.952	1.704	5.901	2.2–4.1
	**neopterin**	2.985 ± 0.641	2.015	4.464	2.3–3.7

UHPLC (ultra-high performance liquid chromatography), SD (standard deviation).

## Data Availability

Data are contained within the article.
